# Reliability and criterion validity of self-measured waist, hip, and neck circumferences

**DOI:** 10.1186/s12874-016-0150-2

**Published:** 2016-05-04

**Authors:** Pamela Barrios, Jennifer Martin-Biggers, Virginia Quick, Carol Byrd-Bredbenner

**Affiliations:** Department of Nutritional Sciences, Rutgers University, 26 Nichol Avenue, New Brunswick, NJ 08901 USA

**Keywords:** Waist, Neck, Hip, Body circumferences, Reliability, Criterion validity, Measurements

## Abstract

**Background:**

Waist, hip, and neck circumference measurements are cost-effective, non-invasive, useful markers for body fat distribution and disease risk. For epidemiology and intervention studies, including body circumference measurements in self-report surveys could be informative. However, few studies have assessed the test-retest reliability and criterion validity of a self-report tool feasible for use in large scale studies.

**Methods:**

At home, mothers of young children viewed a brief, online instructional video on how to measure their waist, hip, and neck circumferences. Afterwards, they created a homemade paper measuring tape from a downloaded file with scissors and tape, took all measurements in duplicate, and entered them into an online survey. A few weeks later, participants visited an anthropometrics lab where they measured themselves again, and trained technicians (*n* = 9) measured participants in duplicate using standard equipment and procedures. To assess differences between self- and technician-measured circumferences, duplicate measurements for participant home self-measurements, participant lab self-measurements, and technician measurements each were averaged and Wilcoxon signed-rank tests conducted. Agreement between all possible pairs of measurements were examined using Intraclass Correlations (ICCs) and Bland-Altman plots.

**Results:**

Participants (*n* = 41; aged 38.05 ± 3.54SD years; 71 % white) were all mothers that had at least one child under the age of 12 yrs. Technical error of measurements for self- and technician- duplicate measurements varied little (0.08 to 0.76 inches) and had very high reliability (≥0.90). Intraclass Correlations (ICC) comparing self vs technician were high (0.97, 0.96, and 0.84 for waist, hip, and neck). Comparison of self-measurements at home vs lab revealed high test-retest reliability (ICC ≥ 0.87). Differences between participant self- and technician measurements were small (i.e., mean difference ranged from −0.13 to 0.06 inches) with nearly all (≥93 %) differences within Bland-Altman limits of agreement and <10 % exceeding the a priori clinically meaningful difference criterion.

**Conclusions:**

This study has demonstrated a simple, inexpensive method for teaching novice mothers of young children to take their own body circumferences resulting in accurate, reliable data. Thus, collecting self-measured and self-reported circumference data in future studies may be a feasible approach in research protocols that has potential to expand our knowledge of body composition beyond that provided by self-reported body mass indexes.

## Background

Anthropometric data collected by self-report surveys are usually limited to height and weight—measurements that are easy, quick, inexpensive, and tend to have a small degree of reporting error in adults [1–7]. These measures typically are used to calculate body mass index (BMI) for the purpose of classifying individuals as underweight, normal weight, overweight, or obese. However, BMI is an indirect measurement of body adiposity and may result in misclassification [8–10]. For instance, those who have a greater proportion of muscle tissue and bone mass, such as athletes and body builders, weigh more and, thus, likely have a BMI that incorrectly indicates weight status. Individuals who are inactive or who have age-related decreases in muscle and bone mass may have a BMI classified as normal weight despite having elevated body fat levels [8]. Additionally, men tend to have more lean muscle mass and less body fat than women even when both have the same BMI [10, 11]. Another limitation of BMI is that it does not reflect body fat distribution (central trunk vs. hips and thighs), which is associated with metabolic disturbances and cardiovascular risks [12–15].

Waist, hip, and neck circumferences are cost-effective, non-invasive, and informative supplementary measurements that could be included on self-report surveys to enhance the usefulness of BMI by serving as indicators of body fatness and fat distribution [15, 16]. Convincing evidence indicates that waist circumference and waist-hip circumference ratio are strongly associated with cardiovascular disease, type 2 diabetes mellitus, hypertension, sarcopenic obesity, colorectal and post-menopausal breast cancer, and, in older adults, declining quality of life and physical activity levels [17–21]. Some have proposed that waist circumference could replace waist-hip ratio and BMI as a single data point to reflect all-cause mortality risk [22]. Others have called for waist and hip circumferences to be routine metabolic and cardiovascular health clinical measures [13, 23] and used as indicators for weight loss interventions [7, 15].

Neck circumference is a relatively new, economical, and practical measure identified as a useful marker for upper body obesity [24, 25]. It correlates positively with metabolic syndrome risk, cardiovascular risk, and elevated blood pressure in children, and pregnancy-induced hypertension [25–31]. In addition, evidence indicates that it is a stronger indicator of elevated serum triglycerides and decreased serum HDL cholesterol (atherogenic dyslipidemia) than BMI and waist circumference in both sexes, making it a useful, non-invasive diagnostic tool [32].

Including circumferences in self-report surveys is worthwhile only if the values reported are accurate. Dutch, overweight workers who were sent a tape measure and written instructions for measuring their own waist circumference had self-reported values that were highly correlated with researcher-measured waist circumference [16]. Other studies using similar methodology also found technician-measured and self-measured circumferences were highly correlated for waist and hip, did not differ significantly, and had no consistent trend across studies in under- or over-reporting [7, 10, 33–40]. No findings could be located to establish reliability of self-measured neck circumferences.

Training materials have been developed to improve circumference measurement accuracy. For instance, English-speaking adults in Scotland and Belgium were given a measuring tape and asked to measure their own waist and hip circumferences using written instructions or training video instructions; those using the training video reported more accurate waist circumferences measurements [41]. Completing a 25-min computer-based training with a reading grade level of 11.7 in a laboratory setting prior to self-measurement resulted in waist circumferences that did not differ significantly between college students and trained staff [37].

Previously published research comparing precision of self-report vs trained-technician measurements indicate self-report measurements may be sufficiently accurate for epidemiological studies [33–35, 38, 42, 43]. The few research studies available suggest that training, especially video instructions, have the potential to improve self-reported waist measurement accuracy [37, 41]. These findings are promising, but their application remains limited for numerous reasons. For example, the instructions (written and video) provided to study participants are generally unavailable beyond the study participants. Additionally, the participant burden (e.g., training time needed and difficulty level of training materials) is beyond what many individuals are willing or able to invest [37] and the ecological value was sacrificed in many studies because training and self-measurements were conducted in a laboratory setting [37, 38, 44].

A key factor limiting application and replication of existing research is the tape measure used. That is, previous studies have relied on tape measures with special characteristics [10, 44] or one mailed to participants [7, 16, 39, 45]—this limitation makes it costly and logistically-difficult to conduct a large scale survey or promote self-measurement as a strategy for self-monitoring of health. In addition, little is known about the reliability of self-measurements over time in any population group [46]. Another limitation of published studies is the statistical procedures used to compare self- and technician-measurements. Many report only correlation coefficients (e.g., Pearson, kappa, ICC), which demonstrate strength of relationship between two raters, but do not reflect inter-rater agreement (e.g., Bland-Altman plots, also called Tukey Mean Difference plots [47]). Of those reporting Bland-Altman plots, no studies of technician- vs self-measurements could be located that applied the array of reporting standards for Bland-Altman analysis of agreement between measurements taken by technicians vs. self [20, 36]. Thus, to overcome limitations of previous research and ascertain the test-retest reliability and criterion validity of a self-report tool feasible for use in large scale studies, this study compared self-measurements of waist, hip, and neck circumferences taken by novice lay people (i.e., mothers of young children) at home after viewing a brief, simple online instructional video and creating a homemade paper measuring tape from a downloaded pdf file to measurements taken by trained technicians using research-grade equipment and standard procedures.

## Methods

The Institutional Review Boards at the authors’ university approved study procedures. All participants gave informed consent.

### Sample

Participants were recruited via announcements posted on community websites and distributed through workplace listservs. Recruitment materials invited individuals to learn to accurately measure their neck, waist, and hips and then have these measurements taken by a trained researcher. Participants received $25 for completing the study. To be eligible for this study, participants had to be women, between 18 and 45 years of age, have at least one child under 12 years of age, and not be pregnant within the past year.

### Development of study tape measure and video

**Fig. 1:**
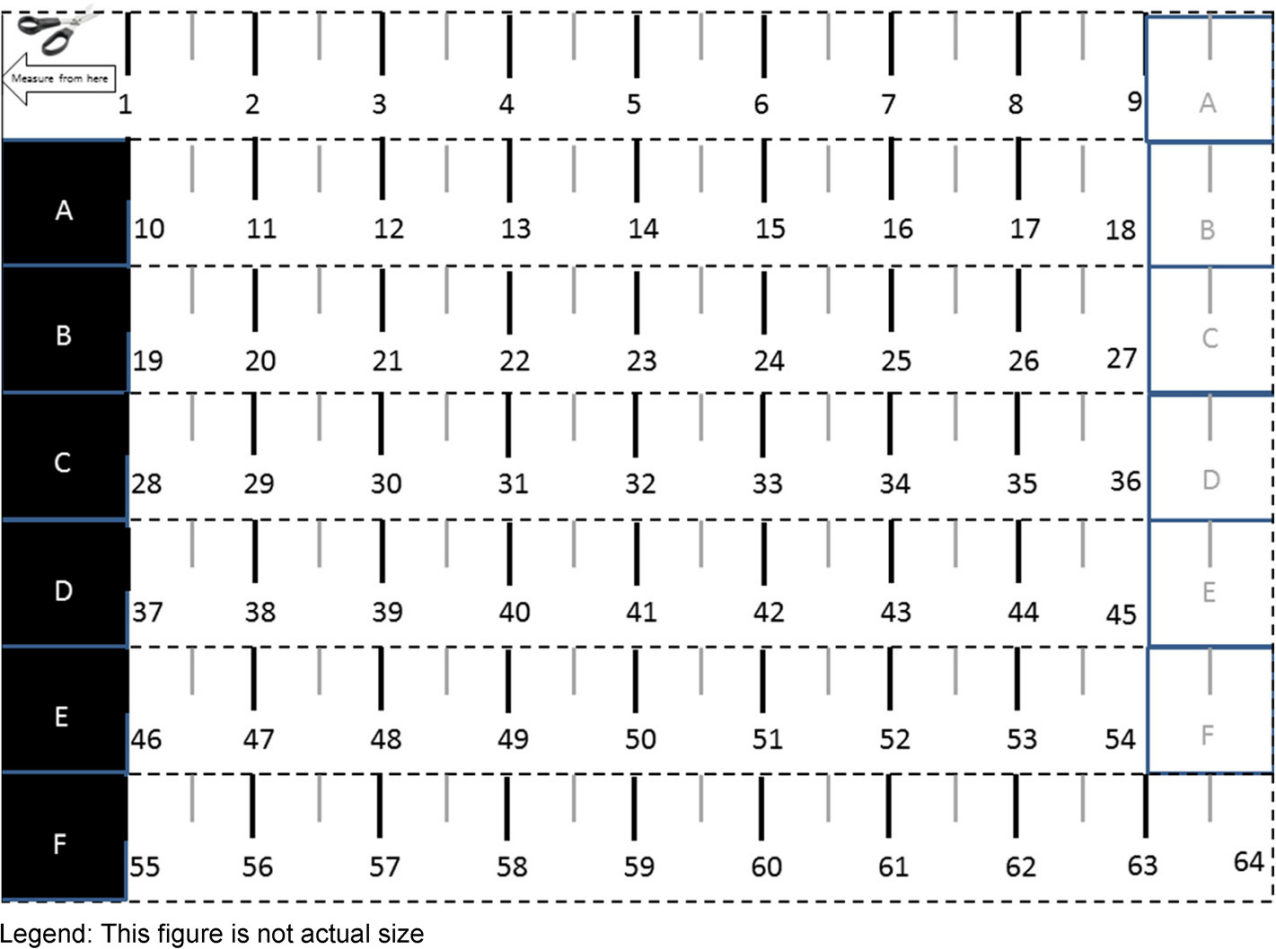
Tape Measure Assembled by Study Participants. Legend: This figure is not actual size

Tape measures that can be downloaded, printed on home printers, and assembled with scissors and tape are commonly used by online clothing companies to ensure ordered clothing will properly fit purchasers. Development of the tape measure for this study began by collecting and reviewing a wide array of online tape measures and assessing them for measurement accuracy, ease of assembly, and clarity of instructions. Existing tape measures were extensively adapted to create the tape measure used in this study; adaptations included developing by clarifying assembly instructions and improving labeling of cutting lines and pieces to be joined by tape (see Fig. 1).

Development of the video began by writing scripts using consumer-friendly terminology. The scripts included instructions for creating the tape measure and taking neck, waist, and hip circumferences. The scripts were reviewed for technical accuracy by a panel of experts in anthropometric measurements and instructional design (*n* = 4) and iteratively refined and shortened. The key points addressed in the video are shown in Table 1. Waist was measured at the level of the belly button (umbilicus) [48–50], hips measured at the level of maximum extension of the buttocks [17, 50], and neck at a point halfway between the collar bone and chin in the middle of the neck [25].

**Table 1:** Key Points Addressed in the Body Measurements Video

• Exact measurements are very important	
• Tape Measure	
• Printing	
• Verifying accuracy	
• Assembly	
• Waist circumference	
• Remove clothing or wear tight fitting clothing	
• How to hold the tape	
• Placement of tape over belly button	
• Keep tape flat and snug against skin	
• Use a mirror to ensure accurate tape placement	
• Inhale then exhale	
• Draw tape in to determine measurement	
• Record measurement	
• Repeat measurement	
• Hip circumference^a^	
• Neck circumference^a^	

^a^Same steps as in waist circumference. Placement for hips was at the fullest part of buttocks. Placement for neck was estimated as the halfway point between the collar bone and chin (at the point of the larynx) with eyes focused straight ahead

Before participants were recruited, the tape measure and video were posted online. The tape measure and video underwent formative cognitive testing with women similar to the study participants, but not included in the study reported here, to verify clarity of information, accuracy of interpretation, and application of the information; it was iteratively refined based on formative testing findings. Subsequently, the tape measure and video were pilot-tested with 7 women recruited in the same way as the study sample and having characteristics similar to those in the study sample, but not in the sample, and again refined.

### Study design

Participants completed an in-home assessment, including self-measures and an online questionnaire (part 1), followed by a clinical visit (part 2). In part 1, participants viewed the less than 9 min instructional video explaining how to measure their own waist, hip, and neck circumferences using the measuring tape they printed out and assembled. Participants were advised to watch the video carefully and as many times as required until they felt sufficiently confident to take their measurements accurately. They also were instructed to pause the video at each of these points to complete the task before proceeding: assemble the tape measure, measure waist, measure hips, and measure neck. The video provided verbal instructions along with photos of women demonstrating the measuring procedure. Participants were instructed to wear minimal and/or snug-fitting clothing, fast for 4 h and void their bladders before taking any measurements, take measurements at the end of a normal expiration, take all measurements in duplicate to the nearest ½-inch, record measurements immediately after taking them, and then enter the measurements into an online survey after all measurements were completed.

The survey also collected participant name, demographic data, height, and weight and evaluated video clarity and ease of constructing the tape measure. Participants were instructed to retain the tape measure.

In part 2, participants visited a campus anthropometrics lab. At the lab, technicians confirmed participants took their measurements at home using the tape they assembled and brought to the lab. The participant-assembled tape measure was labeled and later analyzed for accuracy of assembly. Participants were instructed to fast 4 h before the visit and to wear light, snug clothing. At the lab, participants were instructed to void their bladders, watch the video, and take their measurements in duplicate in the same way they did at home using a commercial measuring tape like those used in home sewing (the home-assembled paper tape measures were not used in the lab to preserve them for later analysis). Trained-technicians, blind to participants’ self-reported measurements taken at home, observed participants while they took and recorded their self-measurements.

Then, technicians measured participants’ circumferences in duplicate using a Gulick tape measure (Country Technology, Inc., Gays Mills, WI) and standard research methods based on the same anatomic landmarks as the participants were instructed to use. Technicians also measured in duplicate heights without shoes to the nearest ¼-inch using a calibrated wall-mounted stadiometer (QuickMedical, Issaquah, WA) and weights to the nearest ¼-pound with a calibrated digital scale (Tanita model TBF-300WA, Arlington Heights, Illinois). At the conclusion of the session, technicians briefly interviewed participants to explore their perceptions of the clarity and ease of following the instructions in the video and to identify suggestions for improvement.

Prior to data collection, research technicians (*n* = 9) were trained to complete study measurements accurately. Technicians reviewed standard anthropometric measurement protocol [51], discussed the protocol with the lead technician, viewed live demonstrations of measurements being taken, and then practiced taking measurements until they achieved a high degree of accuracy compared to the lead technician. The coefficient of inter-observer reliability was above 0.96 for all measurements.

### Data analysis

Analyses were performed using the SPSS for Windows statistical software package version 21.0 (SPSS Inc., Chicago, IL, USA) and Excel (Microsoft, Seattle, WA, USA). Technical error of measurement was calculated for each set of duplicate measurements to assess intra-observer error and reliability [51–54]. To assess differences between self- and technician-measured circumferences, duplicate measurements for participant home self-measurements, participant lab self-measurements, and technician measurements each were averaged and Wilcoxon signed-rank tests conducted. Agreement between all possible pairs of measurements were examined using Intraclass Correlations (ICCs). Statistical significance was set at *P* < 0.05.

Home and lab self-measurements were compared to establish test-retest reliability (repeatability of measurements). In-depth comparisons of participant home self-measurements and technician measurements were conducted because home measurements are analogous to those that participants would self-report in surveys and technician measures can be considered the comparative “gold standard” or measure to establish criterion validity [33]. Analysis procedures for Bland-Altman plots incorporated the array of reporting standards for agreement analysis in laboratory research [20]. These plots graphically illustrate the agreement between participant home self-measurements and technician measurements [47, 55, 56]. The plots include the mean difference (also called bias), limits of agreement (LOA, which are 95 % confidence limits for the bias) calculated using the formula for small samples [57], 95 % tolerance limits for upper and lower LOA (also referred to as 95 % confidence limits for the population) [57], and confidence limits for the bias calculated using standard error of the bias [56].

A comparison of the magnitude of measurement errors between study participants (i.e., untrained lay people) and technicians (i.e., health professionals trained in anthropometrics) was conducted to determine whether self-measurements by untrained lay persons using a self-assembled tape measure were sufficiently accurate for research purposes. A mean difference of ≥ ±10 % was set a priori as the clinically meaningful difference between participants and technicians. This difference was set after scrutinizing previous research for guidance. For example, a review article examining the magnitude of measurement error for waist circumferences taken at various anatomical locations (none included umbilicus) reported that intra-observer and inter-observer measurement error ranged from 0.7 to 9.2 cm (0.28 to 3.62 inches at 2.54 cm per inch) and 1.4 to 15 cm (0.55 to 5.90 inches), respectively, with untrained health professionals tending to have greater measurement error than health professionals trained in anthropometrics [46]. Authors of the review paper concluded it was “difficult to draw conclusions on the magnitude of measurement error [46].” Previous research has noted strong inter-observer differences in waist and hip measurements [46, 51, 58], even when observers were health professionals trained in anthropometrics. Additionally, studies rarely report absolute measurement error (e.g., inches different between observers) [46]. Although no reports of error as a percent of body circumferences could be located and a clinically meaningful difference for inter-observer or intra-observer measurements of waist circumference [46], or other body circumferences, could not be gleaned from the literature, Verweij et al. [46] proposed that a 5 % change in waist circumference measurements taken by trained health professionals may be a clinically relevant short-term change for improvements in health conditions positively associated with waist circumference (e.g., cardiovascular disease). The > ±10 % level was identified as the clinically significant level for this study after considering the inter-observer differences in measurements among trained health professionals reported by others [46, 58], Verweij et al’s [46] “realistic” range of waist circumferences (23.6 inches [60 cm] to 53.15 inches [135 cm]), the current lack of guidance with regard to body circumferences, and examination of studies comparing tests for other measures (i.e., blood glucose, vitamin D, total cholesterol, and triglycerides) which deemed values exceeding approximately 7 to 15 % as clinically significant measurement errors [59–62].

## Results

Participants (*n* = 41) were 38.05 ± 3.54SD years, 71 % white, and 78 % had a bachelor’s degree or higher. As shown in Table 2, the technical error of measurement for home self-, participant lab self-, and technician- duplicate measurements indicated very minor differences (i.e., 0.08 to 0.76 inches) and very high reliability (≥0.90). Table 2 also reports means, ranges, and ICC for measurements. All ICCs comparing participant home vs participant lab, participant home vs technician, and participant lab vs technician met the benchmark for near perfect agreement (i.e., the ICCs fell within the 0.81 to 1.0 range) [63–68]. A comparison of the duplicate technician and self-measurements indicated high measurement repeatability because little difference occurred between the paired measurements for any circumference (i.e., mean difference ranged from −0.13 to 0.06 inches).

**Table 2:** Participant Characteristics and Intra-Class Correlations (ICC) between Participant Self-Measurements and Technician Measurements (*N* = 41)

Measurement	Participants Home Self-Measured	Participants Lab Self-Measured	Technician Measured	Participant Home vs Lab		Participant Home vs Technician		Participant Lab vs Technician	
	Mean ± SD^a^ (range) *TEM/R* ^b^	Mean ± SD^a^ (range) *TEM/R* ^b^	Mean ± SD^a^ (range) *TEM/R* ^b^	ICC	*P* value^c^	ICC	*P* value^c^	ICC	*P* value^c^
Waist Circumference (inches)	35.24 ± 5.73 (26.50–61.00) *0.76/0.90*	34.29 ± 4.85 (26.0–51.5) *0.29/0.98*	34.72 ± 5.69 (26.00–57.63) *0.22/0.99*	.96	.00	.97	.10	.96	.63
Hip Circumference (inches)	40.95 ± 4.79 (33.75–57.00) *0.38/0.97*	40.67 ± 4.66 (33.25–52.75) *0.25/0.99*	41.15 ± 5.80 (33.25–64.25) *0.20/0.99*	.97	.20	.96	.46	.97	.09
Neck Circumference (inches)	13.66 ± 1.19 (11.75–17.00) *0.24/0.95*	13.28 ± 0.93 (11.75–15.75) *0.15/0.98*	12.87 ± 0.88 (11.50–15.00) *0.08/0.99*	.87	.00	.84	.00	.95	.00
Height (inches)	64.32 ± 2.17 (60.00–70.00)	—	64.16 ± 2.27 (60.19–70.72)	—	---	.95	.41	—	---
Weight (pounds)	153.56 ± 39.91 (99.00–315.00)	—	153.32 ± 42.14 (93.81–326.40)	—	---	.99	.32	—	---
BMI	26.08 ± 6.55 (18.11–52.42)	—	26.20 ± 7.12 (17.16–54.52)	—	---	.99	.92	—	---
Waist-to-Hip Ratio	0.86 ± 0.07 (0.73–1.07)	0.84 ± 0.07 (0.68–0.99)	0.84 ± 0.06 (0.74–0.96)	.93	.02	.83	.12	.88	.79

^a^ *SD* standard deviation

^b^ *TEM* (Technical Error Measurement) calculated between duplicate measurements to establish intra-observer measurement accuracy and *R* (reliability) of intra-observer duplicate measurements

^c^Wilcoxon signed-rank test

A comparison of the participant home and participant lab self-measurements was conducted to establish test-retest reliability. The ICCs for these intra-observer measurements were very high (see Table 2). Despite the significant difference between home and lab waist and neck self-measurements, the mean difference was negligible (i.e., 0.95 and 0.38 inches), respectively. The mean difference between home and lab self-measurements equals about 3 % difference for waist and neck circumferences and less than 1 % for hip circumferences which indicate high test-retest reliability.

**Fig. 2:**
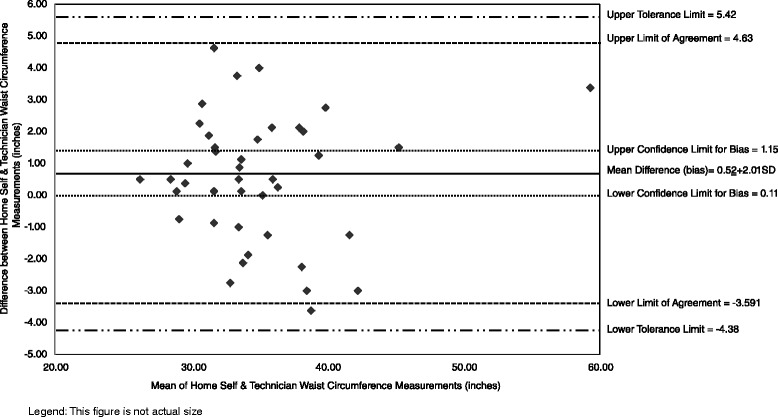
Bland Altman Plots of Waist Circumference of Participant Home Self Measurements and Technician Measurements (*n* = 41). Legend: Diamond shapes are individual observations calculated as home self-measurement – technician measurement; hence positive values indicate self-measurement was larger than technician measurement and vice versa

Figure 2 illustrates the differences between participant home self-measurements and technician waist circumference measurements. The mean difference (bias) indicates that participant waist circumferences were about one-half inch larger than technician measurements; however this measurement did not differ significantly between technician and participant home measurements and did not demonstrate systematic bias. As anticipated, ≥95 % of waist measurement differences fell within the limits of agreement (LOA). A comparison of differences indicated the vast majority (i.e., 93 %) of the participant home waist circumference measurements were ±10 % of technician measurements (i.e., the a priori standard) and thus were not clinically meaningful. The three differences outside the standard differed 12, 12, and 16 %. All three of these cases also had differences outside the standard for one other circumference (1 hip and 2 neck). The upper and lower tolerance limits show the potential agreement expected if similar measures are taken with different samples in the future [56].

**Fig. 3:**
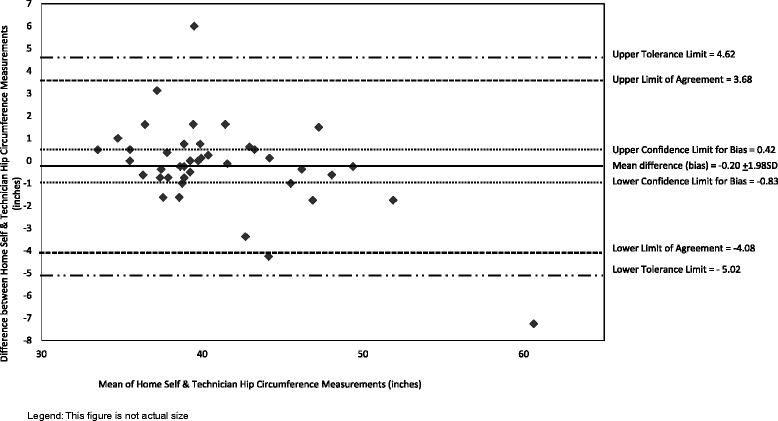
Bland Altman Plots of Hip Circumference of Participant Home Self-Measurements and Technician Measurements (*n* = 41). Legend: Diamond shapes are individual observations calculated as home self-measurement – technician measurement; hence positive values indicate self-measurement was larger than technician measurement and vice versa

The mean difference between home and technician hip measurements was about one-fifth of an inch. Participant home hip measurements did not differ significantly from technician measurements and there was no systematic bias. An examination of the hip measurement differences revealed that 93 % were within the LOA (Fig. 3). As with waist measurements, the vast majority (i.e., 95 %) of the participant home and technician hip circumference measurements were within the a priori standard. The two hip circumferences differences outside the standard differed by 11 and 16 %. Both cases also had differences outside the standard for one other circumference (1 waist and 1 neck).

**Fig. 4:**
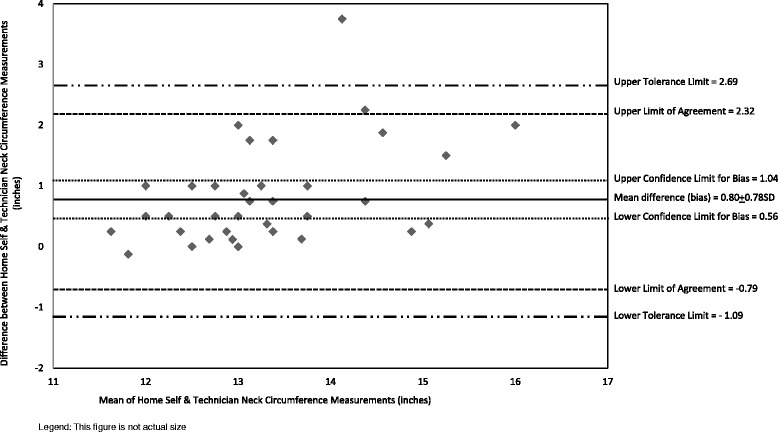
Bland Altman Plots of Neck Circumference of Participant Home Self-Measurements and Technician Measurements (*n* = 41). Legend: Diamond shapes are individual observations calculated as home self-measurement – technician measurement; hence positive values indicate self-measurement was larger than technician measurement and vice versa

The mean difference between home and technician neck measurements showed a slight positive systematic bias, with participant measurements being consistently larger than technician measurements by an average of about eight-tenths of an inch (Fig. 4). Home measurements were significantly greater than technician measurements. However, a comparison of the mean differences with the LOA indicate that 95 % of the differences were within the LOA. Most (i.e., 80 %) participant home and technician neck circumference measurements were within the a priori standard indicating these measurements were not clinically meaningful. The neck circumferences differences that were not within the standard (*n* = 7) differed by 13, 14, 14, 14, 17, 17, and 31 %; all of these values except two differed by 2-inches or less. Two of these cases had measurements outside the standard for one other circumference (both were for waist).

An examination of the tape measures participants made at home indicated that nearly all followed the online instructions and assembled the measuring tapes correctly. Only three participants did not correctly assemble the measuring tape. Their most common error was not taping pieces of the tape measure together at the correct locations; despite this error, measurements from two of these women were very similar to technician measurements whereas the third woman underestimated measures by more than 2 inches. Using a 5-point scale (1 = not easy at all and 5 = very easy), participants rated the ease of making the tape measure 4.6 ± 0.48SD. To further improve ease, participants suggested making the dotted cutting lines darker to help them cut the paper tape straight.

Participants had limited suggestions for refining the instructional video. A few felt more information on how to identify the widest part of their hips was needed beyond the pictures depicting this in the video. A few thought the >9-min video was too long and detailed.

Technician observations of participants when the participants were measuring themselves in the lab indicated that >80 % of mothers completed all self-measurement procedures without errors. Errors observed in some participants were not keeping the tape measure flat, placing the tape measure at incorrect locations on waist or hips, wearing inappropriate clothing or not removing clothing, and incorrectly reading measurements on the tape measure.

## Discussion

The aim of this study was to evaluate the test-retest reliability and criterion validity of self-measurements taken by novice lay persons using a self-assembled tape measure after viewing a brief online instructional video. Results indicate that participants were able to accurately assemble the tape measure and demonstrate proficiency in measuring themselves when observed by lab technicians. The low technical error measurements and high reliability for duplicate measurements demonstrates excellent intra-observer accuracy and reliability. The high ICCs between participant home and lab waist, hip, and neck circumferences indicate that participant self-measurements are highly reliable over time, which is congruent with the limited research reporting reliability of self-measurements [10, 36]. The high reliability indicates that measurements individuals take over time can help them accurately track physical changes that may enable them, their health care providers, and researchers to better realize individuals’ increasing or reducing risk for health conditions associated with high waist and neck circumferences and high waist:hip ratios, such as type 2 diabetes mellitus, cardiovascular disease, and metabolic syndrome [17–19].

The high ICCs between participant home and technician (criterion) measurements for all circumferences indicate measurements made by lay people using paper self-assembled tape measures and a brief online training video are comparable to those of trained health professionals using research-grade equipment and, thus, demonstrate good criterion validity. This finding also suggests that it is feasible to cost-effectively gather accurate self-measurements using a flexible, inelastic paper tape measure self-assembled from a pdf downloaded from the internet for large scale consumer surveys and intervention studies where participants are geographically distant from researchers and, thus, cannot easily visit anthropometric labs for measurement by trained technicians.

The mean differences in waist, hip, and neck circumferences between participants and technicians were small (0.95 inches [2.41 cm], 0.28 inches [0.71 cm], and 0.38 inches [0.97 cm], respectively). A comparison of the mean waist circumference differences reported in other studies of self-measurements (mean difference range = −6.70 to 5.98 cm) indicate the findings from the study reported here (i.e., 0.52 inches or 1.32 cm) are well within this range [7, 10, 33–35, 37–44, 49, 69, 70]. Similarly, studies reporting LOA or SD of mean waist circumference differences thereby permitting LOA calculation, the lower limits ranged from −21.01 to −3.19 cm and the upper range spanned 1.46 to 15.42 cm, or an absolute difference of 4.65 to 33.32 cm. The upper and lower LOA and absolute difference for waist circumference in this study also are well within the values reported by others [7, 10, 34, 35, 37, 38, 41–44, 49, 69]. A similar comparison of mean differences in self-reported hip circumferences published by others [7, 33, 35, 38–41, 43, 44, 69, 70] (mean difference range = −5.90 to 1.19 cm; lower LOA range = −26.09 to −2.29 cm; upper LOA range 1.60 to 14.29 cm; absolute difference of LOA = 6.97 to 40.38 cm) to findings in this study indicate comparable results (mean difference = 1.07 cm, LOA = −10.36 to 9.35 cm; absolute difference 19.71 cm). Also, like other studies, there were no significant differences in mean waist and hip circumferences measured at home and in the lab by technicians [38, 41]. No comparable studies could be found for neck circumference, however the limited research available indicates high agreement for this measure among trained observers [71].

The vast majority of waist and hip circumference self vs. technician measurements were within the a priori standard for differences and, hence, not deemed clinically meaningful. Approximately one-sixth of neck circumference self-measurements differed more than 10 % from technician measurements; this finding, along with the positive bias in neck measurements, indicates a need for improvement. An even tighter agreement between participant and technician circumference measurements would further enhance the utility of self-measurements and may be feasible to achieve. For example, if the a priori standard had been set at < ±5 %, the majority of measurements in this study (i.e., 68, 88, and 78 % for waist, hip, and neck circumferences) would meet this standard.

The lower proportion of waist circumferences (68 %) in the < ±5 % agreement range vs. hip and neck circumferences (88 and 78 %) is of interest. This difference likely is because of the many factors affecting waist circumference throughout the day, including posture, time of day variations in height, fasting vs postprandial state [46, 51, 58, 69, 72], as well as the time gap between home measurements and lab measurements (mean 9.02 ± 6.55 days) and likely differences in phase of the menstrual cycle and associated commonly reported abdominal size changes.

It is important to consider that some differences between technician and participant measurements may be due to the dissimilarity in measurement precision each used. To follow best practices, technicians measured to the nearest ¼-inch. The ½-inch precision level was chosen for participants because previous research revealed that the majority laypersons elected to make self-measurements using ½-inch to 1 inch precision [69]. Additionally, consumers frequently have difficulty accurately interpreting markings denoting fractional quantities when performing measurements [73].

For the most accurate waist and hip measurements, experts recommend standing with feet together, arms at the side, wearing little clothing, being in a fasted state, taking measurements at the end of a normal expiration with the abdomen relaxed, and taking measurements twice and averaging measurements repeatedly until they are within 1 cm of each other [17]. Because the participants in this study were taking their own measurements, they could not keep their arms at their sides or feet together. However, the video did instruct them to wear minimal clothing, read the measuring tape after taking a deep breath in and letting it out, put tension on the tape measure by pulling it gently to be sure it sat flat on the skin but not to pull it tight, and take measurements twice. Additionally, the video repeated instructions for measuring each circumference twice and each time directed them to ensure that the tape measure ran straight across their back (waist), buttocks (hips), or neck and encouraged them to use a mirror to check accuracy of tape measurement placement. Although many protocols do not control for posture and fasting [17, 58], an improvement to the video that should be investigated in future research is to instruct individuals to take waist measurements when standing as erect as possible, after a 4 h or longer fast, and while relaxing their abdomen (not “sucking it in”) [70]. However, the similarity of the home self-measurements and technician measurements suggests participants’ abdomens were relaxed when doing self-measurements. Additionally, Yoon recommended enlisting the assistance of a partner when taking self-measurements because she observed this improved the accuracy of measurements [69].

This study has many important strengths. The tape measure and videos underwent formative cognitive testing by experts trained in qualitative data collection methods and subsequently refined to ensure participant comprehension. Technicians were rigorously trained and had excellent inter-rater reliability scores. In addition, this study is one of the first of its type to include intra-observer technical error measurement and reliability [51–54] as well as test-retest reliability data for self-measurements [10, 36]. A major contribution of this study is establishing the reliability and validity of use a self-assembled tape measure from a downloadable pdf file that is suitable for mass distribution via the Internet at virtually no cost—this innovation has the potential to advance research and promote self-monitoring of body size vis-à-vis personal health. Although creating the tape measure does place some participant burden (e.g., they need to have the appropriate resources, including a computer, Internet, printer, tape), participants in this study reported the tape measure was ease to assemble and did not report any problems. This study is among the few of its type to report confidence intervals for waist, hip, and neck circumferences differences and limits of agreement [34, 43, 44]. Importantly, this study provides the recommended reporting data for Bland-Altman analysis of agreement between measurements taken by technicians vs self. Clinically meaningful levels are rarely reported [20, 21, 56]; this study also is the first known to the authors to propose a clinically meaningful difference in agreement for body circumferences.

This study has numerous strengths, however, the results are limited by the size and homogeneity of the sample (i.e., young women who are mostly white and fairly well educated). Future research should expand the study to males and older adults of varying socioeconomic status and race/ethnicity. Additionally, studies should explore possible training effects (e.g., seeing the video a second time) to ascertain whether it was training effects or other factors (e.g., being observed by technicians) contributing to self-measurements in the lab that were somewhat closer to those of the technician than measurements made at home. Furthermore, an investigation of the effect of providing an interpretation of the measurements to consumers (e.g., health conditions associated with a large waist circumference) on promoting consumer discussions with health care providers would provide insight into the health promotion and motivational utility of self-measurements.

## Conclusions

This study has demonstrated that a simple, inexpensive method for teaching individuals to take their own body circumferences provides reliable and suitably accurate data. Collecting self-measured and self-reported circumference data in research studies is a feasible addition to research protocols and has the potential to expand our knowledge of body composition beyond that provided by just BMI.

### Ethics approval and consent to participate

The Rutgers University Institutional Review Board approved this study and written consent for participation was obtained from all study participants.

### Declarations and availability statement

The dataset supporting the conclusions of this article are available from the corresponding author upon request.
